# The Multifaceted Roles of Macrophages in Rheumatoid Arthritis: From Cytokine Networks to the Discovery of Novel Subsets

**DOI:** 10.3390/ijms27167137

**Published:** 2026-08-09

**Authors:** Xiaowei Yi, Yuhao Liu, Yi Fan, Zhaoqi Zhang

**Affiliations:** Beijing Key Laboratory of Non-Invasive Diagnosis and Immunotherapy of Rheumatic Diseases, Department of Rheumatology and Immunology, Peking University People’s Hospital, Beijing 100044, China

**Keywords:** rheumatoid arthritis, macrophages, cytokine networks, synovial tissue immune microenvironment

## Abstract

Macrophages play a central and multifaceted role in the pathogenesis of rheumatoid arthritis (RA). This review synthesizes current understanding, emphasizing how M1/M2 polarization imbalance drives RA progression. Specifically, M1 promotes synovial inflammation, joint destruction, and pannus formation via pro-inflammatory cytokines (TNF-α, IL-1β, IL-6) and dysregulated angiogenesis. Conversely, functional impairment of anti-inflammatory M2 macrophages contributes to defective immune regulation. Beyond the classical M1/M2 dichotomy, macrophages exert their pathogenic influence through complex networks: activating adaptive immunity by phagocytosis and antigen presentation, secreting cytokines to regulate synovial tissue immune microenvironment and sustaining inflammation by metabolic reprogramming. Critically, heterogeneous macrophage subsets exhibit divergent roles. Recent studies have identified novel populations, such as pro-fibrotic SPP1^+^ macrophages and interferon-responsive STAT1^+^CXCL10^+^ macrophages. Future research may focus on reprogramming macrophage polarization, modulating metabolic pathways, targeting epigenetic regulators, or selectively manipulating specific pro-resolving subsets. The development of multi-target biologics, small molecules, and nanocarrier-based delivery systems could pave the way for personalized RA therapeutics.

## 1. Introduction

The core pathological process of rheumatoid arthritis (RA) arises from loss of immune tolerance, leading to the activation of autoreactive lymphocytes, chronic synovitis, and progressive bone erosion. Approximately 70–80% of patients are seropositive for rheumatoid factor (RF) and anti-cyclic citrullinated peptide antibody (ACPA). Among these patients, ACPA activates the complement system by recognizing citrullinated proteins, directly participating in the initial stage of synovial inflammation [1]. Radiologically, RA presents characteristic marginal bone erosion and synovial enhancement, reflecting the continuous destruction of the joint microenvironment [2].

Macrophages represent the dominant infiltrating cell population in the synovium and act as a central driver of immune network dysregulation. Macrophage-derived pro-inflammatory cytokines such as TNF-α and IL-1β activate fibroblast-like synoviocytes (FLS) to secrete matrix metalloproteinases (MMPs) that degrade cartilage matrix [3]; Meanwhile, macrophages highly express HLA-DR molecules to present autoantigens to CD4^+^T cells, amplifying the pathogenic response of Th17 and Tfh cells [4]; in the chronic inflammatory microenvironment, macrophages polarize to the M1 phenotype and maintain a persistent inflammatory state through the succinate-HIF-1α metabolic axis [5]. Notably, IFN-γ suppresses the immunoregulatory function of synovial PD-L1^+^ macrophages. This suppression further exacerbates immune homeostasis imbalance [6].

T lymphocytes and B lymphocytes synergistically amplify autoimmune damage. Specifically, Th17-derived IL-17A upregulates RANKL expression in mesenchymal cells and promotes osteoclastogenesis [7]. Meanwhile, plasma cells differentiated from B cells secrete ACP-antibodies, which subsequently bind to their cognate citrullinated antigens to form immune complexes that chronically activate macrophages via the Fcγ receptor pathway, thus perpetuating a pathological circuit [8]. During persistent inflammation, FLS acquire an abnormal hyperproliferative and invasive phenotype characterized by enhanced Cadherin-11-mediated aggregation, elevated CCL2/CCL5 secretion, and activated Wnt5a/Ror2 signalling that drives pathological pannus growth [9,10].

Beyond cellular crosstalk, the progression of RA involves stage-specific pathological alterations. In the initiation phase, the cytosolic DNA sensor AIM2 recognizes DAMPs and forms the AIM2 inflammasome complex, which activates caspase-1; caspase-1 then cleaves pro-IL-1β into its mature form, thereby promoting neutrophil infiltration [11]. In the chronic inflammatory phase, synovial hypoxia induces HIF-1α-dependent neovascularization; mitochondrial dysfunction triggers reactive oxygen species (ROS) overproduction; and TNF-α/IL-6 signalling impairs Treg cell function [12]. In the terminal stage, bone destruction is marked by an imbalance in the RANKL/RANK/OPG axis, where hyperactivated osteoclasts drive irreversible subchondral bone erosion [13].

Multiple systemic factors modulate disease progression. Gut dysbiosis such as enrichment of *Prevotella*, promotes ACPA production by elevating mucosal IL-17A [14,15]. Global genomic DNA hypomethylation in synovial cells triggers excessive transcription of inflammatory genes, including TNFa and IL6 [16]. In macrophages, accumulation of α-ketoglutarate sustains pro-inflammatory epigenetic memory via TET2 demethylase activation [3]. Collectively, these mechanisms lead to differences in clinical treatment responses. For example, patients with positive autoantibodies show more significant efficacy in B cell depletion therapy [17].

Current therapeutic strategies are increasingly focused on multi-targeted interventions. Bispecific antibodies provide simultaneous blockade of IL-6R and TNF to counteract cytokine network compensation [18]. Nanocarrier-based gene silencing enables targeted downregulation of key transcription factors such as IRF5 in synovial cells. Future research should integrate single-cell multi-omics to dissect the dynamic macrophage–FLS–lymphocyte network [19] and explore intervention targets in metabolic reprogramming and epigenetic regulation [20]. These efforts will pave the way toward more precise and personalized treatment of RA.

## 2. M1/M2 Polarization Imbalance Leads to RA Progression

The classical M1/M2 paradigm is a simplified classification derived largely from in vitro experimental conditions. In RA synovium, macrophages exist along a functional continuum shaped by tissue niche, disease stage, and local signals. We use this framework as a reference, while recognizing that recent single-cell studies have revealed far greater heterogeneity than the M1/M2 dichotomy captures.

### 2.1. A. Increased M1 Polarization Induces Inflammatory Responses

In rheumatoid arthritis (RA), aberrant activation of pro-inflammatory M1 macrophages critically drives joint destruction. These cells are markedly enriched in the synovial inflammatory milieu, and their infiltration density correlates positively with disease activity (DAS28) [5,21,22]. M1 macrophages instigate tissue injury via multifaceted pathways: secreted mediators such as TNF-α and IL-1β trigger cascading NF-κB and MAPK signalling, amplifying the release of IL-6 and IL-23, which in turn promote Th17 differentiation and osteoclast activation, culminating in irreversible bone erosion [5,21,22]. Notably, the maturation and secretion of IL-1β itself depend on NLRP3 inflammasome-mediated caspase-1 activation, and NLRP3 signaling has been shown to potentiate Th17 cell differentiation through IL-1β-dependent pathways. Thus, the NLRP3–IL-1β axis acts as an upstream amplifier of the pro-inflammatory and Th17-skewing circuit in RA [23]. Multiple studies have shown that TNF-α levels are significantly elevated in the serum and synovial fluid of RA patients, and these levels are associated with radiographic progression [22,24].

Concomitantly, fatty acid-binding protein 4 (FABP4), specifically released by M1 macrophages, drives pathological pannus overgrowth via endothelial VEGFR2 and downstream NF-κB activation [25]. Histological evidence reveals markedly increased synovial vascularity in RA relative to osteoarthritis; these aberrant vessels not only invade cartilage but also directly activate osteoclasts through RANKL secretion, establishing a self-perpetuating “pannus–bone erosion” cycle [5,21]. Regarding cartilage destruction, M1 cells abundantly express matrix metalloproteinases (MMP-3, MMP-13) and aggrecanase (ADAMTS-5), which degrades collagen type II and proteoglycan matrix. Their conditioned medium has been shown to enhance chondrocyte apoptosis and suppress COL2A1 expression, a critical gene for collagen synthesis [5,6,21].

Furthermore, the hypoxic synovial microenvironment stabilizes hypoxia-inducible factor-1α (HIF-1α), which upregulates glycolytic machinery (GLUT1, HK2, PFKFB3) and leads to elevated synovial lactate. Concurrently, HIF-1α inhibits mitochondrial respiration and sustains pro-inflammatory gene transcription via a positive feedback loop with NF-κB [21,26,27].

### 2.2. B. Decreased M2 Leading to Defects in Anti-Inflammatory Functions

**Figure 1:**
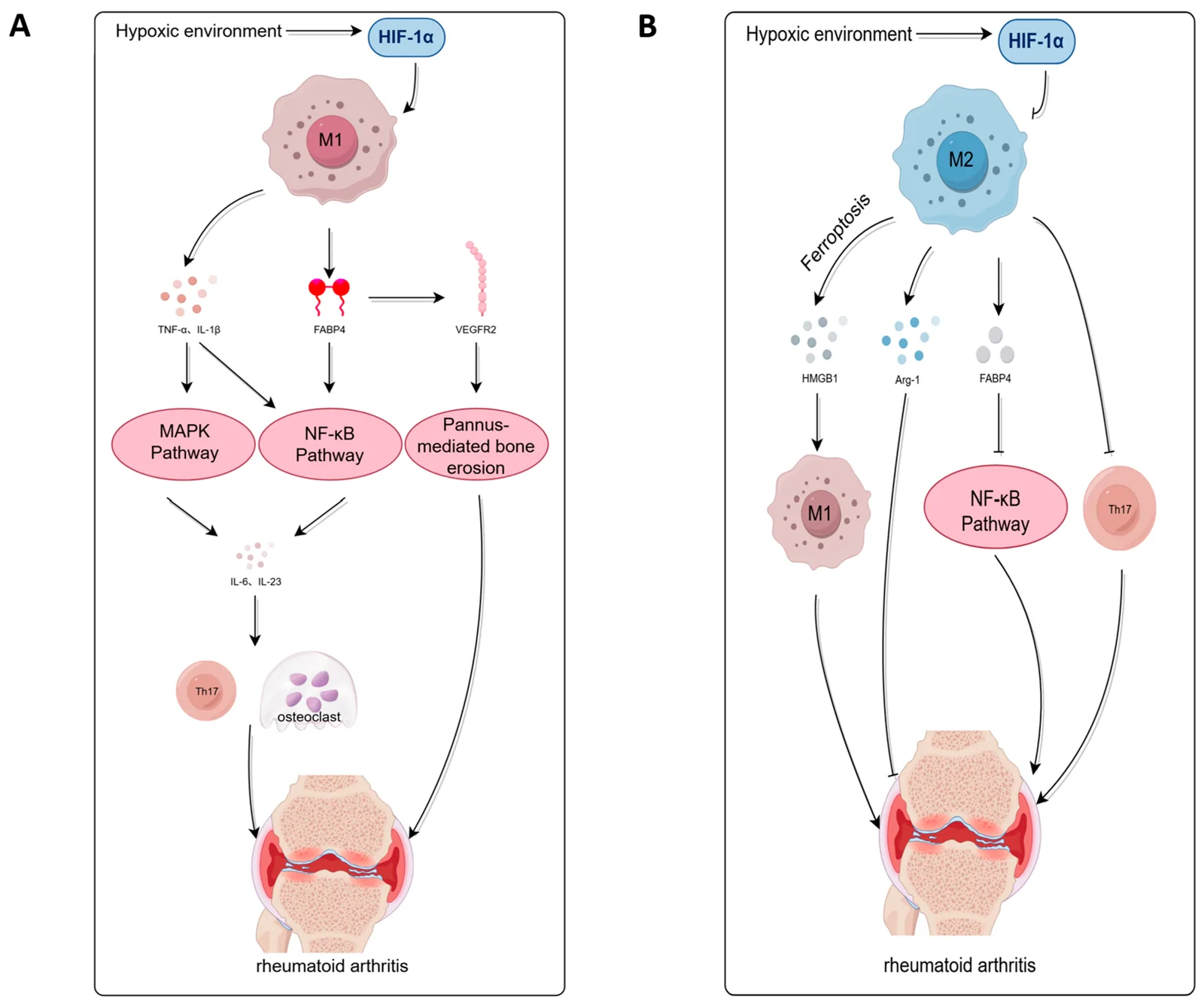
The mechanisms of M1/M2 macrophages in the pathogenesis of rheumatoid arthritis. (**A**) M1 macrophages in RA Hypoxia raises HIF-1α, expanding M1 pool; TNF-α/IL-1β trigger MAPK/NF-κB → IL-6/IL-23 → Th17/osteoclast activation and joint destruction. FABP4-VEGFR2 axis fuels pannus and erosion. (**B**) M2 macrophages in RA: Hypoxia-HIF-1α blunts M2 polarization; M2 ferroptosis releases HMGB1 that amplifies M1, while Arg-1/FABP4 drop and Th17 restraint wanes. M2 loss accelerates RA. In this figure, arrows (→) indicate secretion or promotion of secretion of the indicated molecules, while blunted arrows (⊣) indicate inhibition.

The functional deficiency of anti-inflammatory M2-type macrophages results in profound impairment of immune homeostatic and tissue-repair mechanisms in RA (Figure 1). Synovial M2 cells are markedly diminished in proportion, and their dysfunction manifests as three interrelated deficits [6,22,24]: (1) compromised secretion of anti-inflammatory cytokines, failing to restrain pro-inflammatory mediator production and NF-κB activation, thereby promoting synovial fibroblast aggressiveness. (2) defective tissue repair, owing to reduced arginase-1 (Arg-1) expression, which correlates inversely with cartilage damage. (3) dysregulated immune modulation, with impaired Treg induction and consequent Th17 expansion, culminating in disturbed bone immune equilibrium [6,22,24].

At the molecular level, M2 deficiency arises from multifactorial causes. Synovial iron overload accelerates GPX4 ubiquitination and degradation, provoking lipid peroxidation and ferroptosis in M2 cells [28,29]. Concurrently, HMGB1 released from dying cells engages TLR4 on M1 cells and downstream STAT3 signaling, perpetuating a self-reinforcing cycle of “M2 loss → M1 activation.” In signal transduction, IL-4-driven STAT6 phosphorylation is attenuated, while aberrant LXR activation suppresses the anti-inflammatory transcriptome via MAFB downregulation [25,30,31]. Metabolic reprogramming further diminishes M2 marker expression. This is driven by two parallel mechanisms. First, HIF-1α inhibits CPT1A. Second, NAMPT overexpression suppresses PPARγ through the NAD^+^/SIRT1 axis [25,30,31].

Histopathologically, synovial CD163^+^ M2 cell density is substantially lower than in healthy controls, and this reduction parallels the radiographic progression of joint destruction [6,24]. Functional tests further confirm that peripheral blood monocytes from RA patients exhibit a severely impaired capacity for IL-4-driven M2 polarization, as reflected by markedly reduced CD206 expression relative to healthy controls, pointing to an essential defect in systemic immune regulatory function [24].

## 3. Macrophages Drive the Pathological Process of RA Through Cytokine and Microenvironment Regulation

### 3.1. A. Phagocytosis and Antigen Presentation

In the pathogenesis of rheumatoid arthritis (RA), macrophages profoundly participate and drive the disease’s pathological processes through their key phagocytic and antigen presenting functions. As an important component of the innate immune system, macrophages maintain tissue homeostasis by phagocytosing apoptotic cells, immune complexes, and pathogens. They also connect the adaptive immune responses through antigen presentation, thus playing a core role in chronic inflammation and joint destruction in RA [2,5].

Macrophages display context-dependent functional plasticity in the RA joint microenvironment through phagocytosis mediated by Fc receptors and complement receptors. In the rheumatoid synovium, macrophage uptake of immune complexes and post-translationally modified autoantigens engages TLR/FcγR signaling to elicit IL-1β–driven pro-inflammatory cytokine release, thereby amplifying synovial inflammation [32,33,34]. It is particularly noteworthy that the hypoxic environment of RA lesions can maintain the phagocytic function of macrophages [35] yet significantly inhibits their MHC-I antigen-presenting ability, affecting the activation of CD8^+^ T cells [35,36]. This functional shift in the hypoxic microenvironment is generally conducive to maintaining a pro-inflammatory state and may be an important factor leading to the dysregulation of the immune response in the RA synovium.

As the main antigen-presenting cells, synovial macrophages perform a crucial antigen presenting function in RA [2]. After phagocytosing and processing autoantigens, they present antigenic peptides to CD4^+^ T cells via MHC-II molecules, thereby activating T cell immune response. Meanwhile, macrophages can promote B cell activation through antigen presenting and cytokines secreting [2]. This process leads to T cell-dependent B cell activation, promoting B cell differentiation into plasma cells and the production of pathogenic autoantibodies. The immune complexes formed by these autoantibodies deposit in the joints, causing tissue damage through complement activation, and at the same time can be recognized and phagocytosed by macrophages, forming a vicious cycle of inflammatory amplification [5,33].

Macrophages play a core role in the pathogenesis of RA through two main pathways: phagocytosis and antigen presentation. Their overactive phagocytic function amplifies the inflammatory responses in a specific microenvironment. As key antigen-presenting cells, their functional abnormalities or overactivation directly connect and enhance the adaptive immune response mediated by T and B lymphocytes, driving autoantibody productions and persisting inflammation. These two intertwined pathways jointly promote chronic inflammation, synovial hyperplasia, and progressive joint destruction in RA, establishing macrophages’ central position in RA pathological mechanisms [2,5,10,20,32,33,34,35,36,37,38].

### 3.2. B. Pathogenic Mechanism of Cytokine Networks

In the pathological process of rheumatoid arthritis, macrophages play a core driving role through a complex cytokine network. As an important member of the innate immune system, macrophages polarize to form different functional phenotypes (proinflammatory M1 cells and anti-inflammatory M2 cells), and the imbalance of their homeostasis (dominance of M1 cells) leads to immune dysregulation, exacerbating synovitis and joint damage [5,20,32].

M1-type macrophages secrete a large number of pro-inflammatory factors, including TNFα and IL-6, forming a self-reinforcing positive feedback loop. The activation of the SLAMF7 receptor can induce the expression of TNF-α, further amplifying inflammatory cytokine cascades [39,40]. As a key pathogenic factor of RA, IL-6 directly promotes the persistence of chronic inflammation, while antibody therapy targeting IL-6 can effectively block this pathway [41,42]. At the same time, IFN-γ drives a “hyperactivated” state by regulating the expression of the SLAMF7 receptor on the surface of macrophages, thereby promoting the pro-inflammatory gene program [39].

Macrophages form a complex interaction network with other cells in the synovium. TNF-α secreted by M1 macrophages induces tumor-like invasive behavior in synovial fibroblasts [10,17], while mediators released by FLS such as IL-1β in turn activate macrophages [43]. Through the ADP/P2Y signaling pathway, inflammatory macrophages exacerbate neutrophil-mediated joint damage [44]. Additionally, TNF-α promotes the differentiation of Th17 cells, and IL-17 secreted by Th17 cells further activates macrophages, forming a pro-inflammatory axis [45,46].

Macrophage polarization is precisely regulated by signaling pathways such as Notch, JAK/STAT, NF-κB, and MAPK [5]. JAK inhibitors effectively suppress macrophage activation and alleviate arthritis symptoms [47,48]. Metabolic reprogramming is another key mechanism: M1 macrophages rely on glycolysis whereas M2 macrophages depend on oxidative phosphorylation, with metabolic disorders exacerbating M1 polarization [5]. Itaconic acid, a macrophage-specific anti-inflammatory metabolite, is increased in the synovial fluid of RA patients, and its production disorder may promote the development of inflammation [3].

In summary, macrophages construct a complex inflammatory network by secreting cytokines such as TNF-α, IL-6, and IL-1β, and synergistically drive synovial inflammatory damage with FLS, T cells, etc. (Table 1). Targeting the balance of macrophage polarization, key receptors, and metabolic pathways provides a new therapeutic direction for intervening in the pathological process of RA [5,37].

### 3.3. C. Macrophage-Derived miR-100-5p Remodels the Joint Microenvironment via mTORC1 Activation

In the pathological process of rheumatoid arthritis, macrophage-mediated delivery of miR-100-5p to synovial fibroblasts via extracellular vesicles constitutes a core mechanism driving joint microenvironment remodeling. Studies demonstrated that in the collagen-induced arthritis model, small extracellular vesicles (sEVs) secreted by bone marrow-derived macrophages are enriched with miR-100-5p. After these vesicles are effectively taken up by RA-FLS, they significantly promote the proliferation of synovial cells and the release of inflammatory factors by activating the mammalian target of rapamycin (mTOR) signaling pathway [52]. Mechanistically, miR-100-5p specifically activates the mTORC1 signal by inhibiting DEPTOR, a key mTOR pathway negative regulator, thereby driving the tumor-like behavior of RA-FLS, including abnormal proliferation and enhanced invasiveness [52,53]. Experimental confirmation shows that overexpression of the miR-100-5p mimic in RA-FLS can partially reverse the pro-inflammatory effect of BMDM-sEVs, verifying the direct regulatory effect of this axis [52]. Animal models further show that the defective signal of miR-100-5p delivered by macrophages through sEVs exacerbates the activation of synovial fibroblasts, promote joint erosion and angiogenesis, and ultimately lead to the progression of RA [52,54]. This finding reveals that the cell crosstalk between macrophages and RA-FLS is the core driving force for the chronicity and tissue destruction of RA [10,52]. In conclusion, the macrophage-sEVs-miR-100-5p/mTOR axis precisely regulates the malignant phenotype of RA-FLS, remodels the joint microenvironment, and accelerates the pathological process of RA, providing new ideas for the development of intervention strategies targeting sEVs miRNA delivery or the mTOR signaling pathway [52,53].

## 4. Heterogeneous Roles of Tissue-Resident and Myeloid-Derived Macrophages

### 4.1. A. Protective Function of Tissue-Resident Macrophages Subsets

**Figure 2:**
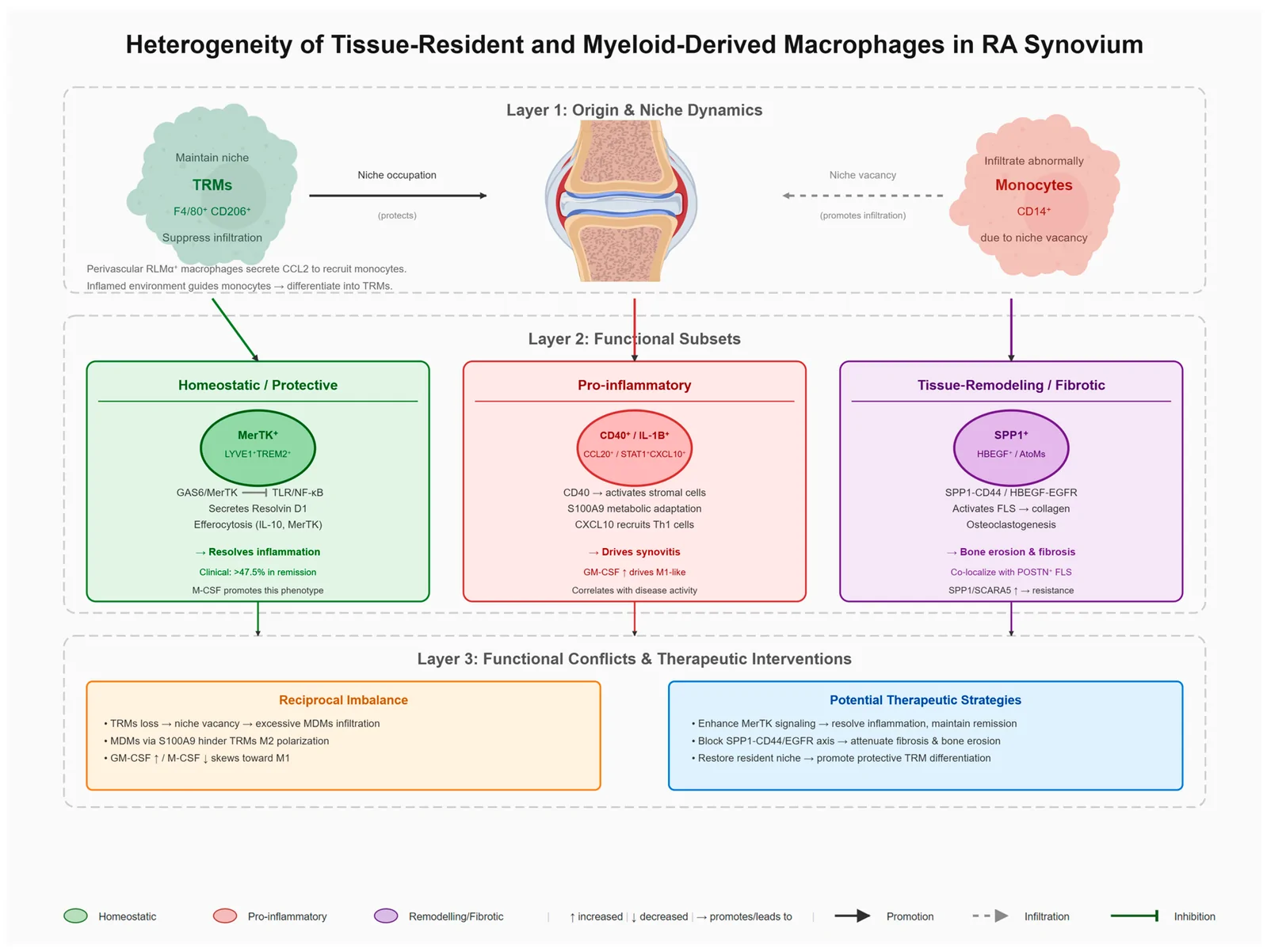
Schematic overview of synovial macrophage heterogeneity in RA. Layer 1: TRMs (F4/80^+^CD206^+^) maintain niche occupancy to limit monocyte infiltration; niche vacancy promotes pathological monocyte influx. Layer 2: three functional subsets—homeostatic/protective (MerTK^+^, green), pro-inflammatory (CD40^+^/IL-1B^+^/STAT1^+^, red), and tissue-remodeling/fibrotic (SPP1^+^/AtoMs, purple). Key clinical correlates: MerTK^+^ > 47.5% in remission; SPP1/SCARA5 ↑ linked to treatment resistance. Layer 3: reciprocal imbalance between subsets drives chronic inflammation; therapeutic strategies include MerTK agonism, SPP1 blockade, and niche restoration. Solid arrows, promotion/differentiation; dashed arrows, infiltration/migration; T-bars, inhibition. Created with BioGDP.com [56]. The ↑ indicates increased level or elevated expression in RA vs. HC. The ↓ indicates decreased level or reduced expression in RA vs. HC. The → indicates promotes. The ┤ indicates inhibits.

Under homeostasis, tissue-resident macrophages inhibit chronic inflammation by occupying specific niches (Figure 2). Vacancy of these niches triggers abnormal monocyte infiltration, exacerbating arthritis; niche supplementation enables normal monocyte differentiation into protective tissue-resident macrophages, thereby suppressing arthritis. This indicated that synovial tissue-resident macrophages (F4/80^+^) constitute a barrier to inhibit chronic inflammation, and their deficiency leads to the exacerbation of arthritis [55].

Perivascular RLMα^+^ macrophages secrete CCL2, which helps to mainly recruit monocytes to the synovial stroma during the onset of antigen-induced arthritis. The inflamed synovial environment guides the recruited monocytes to differentiate into tissue-resident macrophages [57]. PD-L1^+^ macrophages express MerTK and IL-10 and inhibit inflammation through efferocytosis [6].

In contrast to the generally decreased M2:M1 macrophage ratio observed in active rheumatoid arthritis synovitis, in a subset of early, treatment-naïve RA patients with lower baseline disease activity (PtC2), this ratio is significantly increased within the synovial lining layer and inversely correlates with DAS28CRP. The M2 marker CD206, expressed by tissue-resident macrophages, is thought to play a critical role in maintaining synovial tissue homeostasis, suggesting that these lining-layer macrophages may exert a protective role through M2-type polarization, potentially delaying disease progression in this specific subgroup [58].

Tissue-resident macrophages are the first line of defense against pathogens and maintenance of tissue homeostasis. They develop from yolk sac progenitor cells in the embryonic stage, form a stable spatial and functional relationship with tissue cells, and persist in adults [59].

### 4.2. B. Pathogenic Factors of Myeloid-Derived Macrophages

In the RA synovium, myeloid-derived monocytes infiltrate abnormally due to the vacancy of the resident macrophage niche, differentiate into pro-inflammatory M1-like macrophages, release destructive cytokines, and exacerbate arthritis [32,49,55]. The dominant CD40^+^CD206^+^CD163^+^ macrophage population in the RA synovium is related to disease activity and treatment response (Figure 2) [60]. Among them, the IL-1B^+^CCL20^+^ and SPP1^+^MT2A^+^ macrophage subsets are early pathogenic myeloid subsets, which mediate the pro-inflammatory response of stromal cells by activating CD40 signals [60]. Monocyte-derived macrophages undergo metabolic adaptation in the glucose-deficient environment of the synovium, continuously producing pro-inflammatory factors, and maintaining their immunosuppressive phenotype through the S100A9 protein, driving joint destruction [33,61,62].

### 4.3. C. Functional Conflict of Heterogeneous Subsets

The imbalance of their ratio is the key to the persistence of inflammation [5,20,32,58]. GM-CSF induces monocyte-derived macrophages to differentiate into the proinflammatory M1 phenotype, while M-CSF promotes anti-inflammatory M2 differentiation. The high expression of GM-CSF, which is positively correlated with disease activity, and the low expression of M-CSF in the RA synovium exacerbate M1 polarization and disrupt homeostasis (Figure 2) [51].

Monocyte-derived macrophages can differentiate into arthritis-associated osteoclastic macrophages (AtoMs) to drive bone destruction [63], and can also differentiate into PDL1^+^ macrophages to inhibit inflammation through MerTK and IL-10 [6,61]. Tissue resident macrophages limit monocyte infiltration by maintaining the niche [55]; conversely, monocyte-derived macrophages hinder M2 polarization through the S100A9-dependent pathway and interfere with inflammation resolution [60,64]. Targeted interventions for the above heterogeneity, such as remodeling macrophage polarization and restoring the resident niche, are potential therapeutic strategies [2,20,48].

### 4.4. D. Novel Macrophage Subsets

Recent studies integrating single-cell transcriptomics, spatial transcriptomics, and multi-omics data have systematically identified several macrophage subsets beyond the classical framework and clarified their temporal and spatial regulatory mechanisms in the disease process (Figure 2). MerTK^+^ macrophages have been identified as key populations for maintaining synovial homeostasis and achieving sustained remission after drug dose reduction. This subset expresses both LYVE1 and TREM2, localizes in the synovial lining layer, and exhibits potent phagocytic and inflammation-resolving functions. By sensing local GAS6 signals through the MerTK receptor, it can inhibit the proinflammatory program driven by the TLR/NF-κB axis and secrete resolvins (such as Resolvin D1) to induce the transformation of synovial fibroblasts to the repair phenotype [65]. Clinical cohort studies show that the proportion of MerTK^+^ macrophages in the synovium of patients in remission patients is significantly increased; if this proportion drops below 47.5%, the risk of recurrence after drug withdrawal increases significantly, suggesting that it can be used as a tissue-resident biomarker for predicting the persistence of remission [65]. SPP1^+^ macrophages, with osteopontin as the core marker, exhibit dual proinflammatory and pro-fibrotic properties. This subset is often co-expressed with HBEGF and continuously activates synovial fibroblasts through the SPP1CD44/HBEGF-EGFR axis, promoting invasiveness and collagen deposition [66]. Spatial multi-omics studies on the skin of systemic sclerosis (SSc) further confirm that SPP1^+^ macrophages and POSTN^+^ fibroblasts are spatially co-localized, jointly forming a pro-fibrotic niche, and the increase in the SPP1/SCARA5 ratio is related to treatment resistance, suggesting its conservative role in cross-disease fibrotic pathways [67].

In addition, STAT1^+^CXCL10^+^ macrophages represent the interferon-responsive subset, which plays an important role in Th1 cell chemotaxis and chronic inflammation maintenance [66]; while IL1B^+^FCN1^+^HBEGF^+^ macrophages are closely related to early invasive lesions and bone destruction [66]. These subsets establish multi-layered regulatory circuits with fibroblasts, T cells, and B cells via cell–cell communication networks, driving RA synovial inflammation heterogeneity. The detailed analysis of RA synovial macrophage subsets breaks through the M1/M2 dichotomy and reveals functional modules represented by MerTK^+^, SPP1^+^, and CXCL10^+^. Targeting the MerTK signal to enhance inflammation resolution or blocking the SPP1-mediated fibrotic pathway is expected to provide new intervention nodes and biomarkers for the precise treatment of RA (Table 2).

## 5. Summary and Future Prospects

The pathogenesis of rheumatoid arthritis involves extensive, complex crosstalk between macrophages and other immune cells and stromal cells. However, it is imperative to critically revisit the conceptual framework that underpins much of our current understanding of macrophage biology in RA. The classical M1/M2 polarization paradigm, while pedagogically useful, has been increasingly challenged by high-resolution omics technologies. Accumulating evidence from single-cell RNA sequencing and spatial transcriptomics has unequivocally demonstrated that synovial macrophages in RA do not exist as discrete M1 or M2 populations but rather occupy a continuum of activation states, shaped by local niche signals, disease stage, and metabolic conditions [2,5,66]. These studies have identified novel subsets, such as MerTK^+^ pro-resolving macrophages and SPP1^+^ pro-fibrotic macrophages, which display transcriptional programs that do not align with the traditional dichotomy [65,66]. Moreover, the functional plasticity of macrophages is influenced by hypoxia, metabolic reprogramming, and crosstalk with synovial fibroblasts and lymphocytes, further complicating any binary classification [21,26,27]. Therefore, while we have utilized the M1/M2 nomenclature throughout this review for its heuristic value, we recognize that it is an oversimplification. A more nuanced view—one that appreciates the multidimensional spectrum of macrophage functional states—is essential for understanding the pathogenic heterogeneity of RA and for designing precision therapies that target specific subsets or functional pathways rather than broadly modulating polarization. Future studies should leverage single-cell multi-omics and spatial approaches to dissect the dynamic interplay among macrophage subsets and their stromal partners, with the goal of identifying actionable targets for personalized medicine.

Importantly, cross-disease advances offer concrete hypotheses for macrophage centered research in RA. In systemic lupus erythematosus, dysregulated regulated cell death programs directly drive aberrant immune activation [68], while mitochondrial dysfunction not only amplifies ROS-mediated tissue injury but also reprograms immune-cell metabolism [69,70]. Complement-targeted interventions have demonstrated therapeutic benefits across multiple autoimmune conditions [71], supporting the rationale for exploring complement inhibition to modulate macrophage activation and cytokine cascades in RA. In parallel, recent insights into the spatio-temporal control of autophagosome-lysosome fusion [50,72,73] provides a mechanistic framework for how autophagy in macrophages and synovial fibroblasts may regulate chronic inflammation and joint remodeling.

Future research directions should focus on the following aspects: first, using spatial transcriptomics and multi-modal imaging technology to further analyze the spatiotemporal interaction network between macrophages and other cells in the synovial tissue; second, developing intervention strategies targeting macrophage metabolic reprogramming; third, exploring therapeutic methods based on macrophage specific epigenetic regulation; fourth, promoting personalized treatment strategies, selecting the most suitable biological agents or small molecule drugs based on individual patient macrophage subset profiles. Finally, by integrating basic research and clinical data, it is expected to realize the early diagnosis, dynamic monitoring, and precise treatment of RA, and improve the long-term prognosis of patients.

## Figures and Tables

**Table 1:** The influence and mechanism of action of cytokines secreted by M1 macrophage and M2 macrophage on RA.

Cytokine	Secretory Cell	Effect on RA	Mechanism of Action	Clinical Evidence
TNF-α	M1 macrophage	Promoting	Activates NF-κB/MAPK pathways, promotes FLS activation, osteoclast differentiation, and angiogenesis [38,39]	Serum and synovial fluid levels are significantly elevated and correlate with radiographic progression [22,24]
IL-6	M1 macrophage	Promoting	Promotes Th17 differentiation, production of acute-phase proteins, and maintenance of chronic inflammation [41]	Anti-IL-6 therapies (e.g., sarilumab, olokizumab) effectively block this pathway [41,42]
IL-10	M2 macrophage	Dual	IL-10 inhibits signaling pathways such as NF-κB, reducing the production of pro-inflammatory cytokines like TNF-α and IL-1β, and downregulates GM-CSF and FcγR expression, thereby mitigating inflammatory responses [49]	Its anti-inflammatory function is impaired in RA, contributing to defective immune regulation and persistent inflammation [24]
IL-13	M2 macrophage	Dual	IL-13 inhibits signaling pathways such as NF-κB, reducing the production of pro-inflammatory cytokines, and may promote the synthesis of tissue repair-related proteins [49]	-
IL-15	M1 macrophage	Promoting	IL-15 stimulates the activity of immune cells such as T cells and NK cells, promoting the production of pro-inflammatory cytokines like IL-1β, TNF-α, IL-8, and MCP-1, thereby forming a self-sustaining pro-inflammatory loop [49]	-
IL-18	M1 macrophage	Promoting	IL-18 is secreted by CD68^+^ macrophages in RA synovial tissue and can enhance the production of IFN-γ, TNF-α, GM-CSF, and NO by synovial cells, promoting inflammatory responses and bone destruction [49]	Elevated in RA synovial tissue and associated with inflammatory and erosive changes [49]
IL-1β	M1 macrophage	Promoting	Activates the NLRP3 inflammasome, promotes IL-17 secretion, and enhances FLS invasiveness [43]	Synovial fluid levels are raised and correlate with FLS-mediated cartilage degradation [43]
TGF-β	M2 macrophage	Dual	Promotes fibrotic repair, but may also promote Th17 differentiation [5]	Implicated in synovial fibrosis; its pro-Th17 activity may exacerbate inflammation [5]
IL-12/IL-23	M1 macrophage	Promoting	Promotes Th1/Th17 differentiation and maintains chronic inflammation [50]	-
IFN-γ	Th1/M1	Promoting	Enhances M1 polarization, promotes antigen presentation, and inhibits M2 polarization [46]	Drives M1-polarisation and contributes to chronic inflammatory maintenance [46]
GM-CSF (granulocyte-macrophage colony-stimulating factor)	M1/M2 macrophage	Promoting	Activates macrophages and neutrophils, promoting inflammatory responses. It also induces the production of IL-6 and IL-23, further activating T cells and their differentiation into Th17 cells, thereby maintaining the inflammatory cycle [49]	Expression levels positively correlate with disease activity (DAS28) [51]
NO	M1 macrophage	Promoting	Increases the production of pro-inflammatory cytokines such as TNF-α, thereby promoting synovial inflammation and influencing bone remodeling [49]	-

**Table 2:** Functions of Heterogeneous Macrophages in the RA Joint Microenvironment.

Category	Macrophage Subset	Key Markers/Functions	Role in RA	Clinical Evidence
Protective Macrophages	F4/80^hi^ MHCII^−^ synovial tissue-resident macrophages	Niche occupancy, suppression of aberrant monocyte infiltration, maintenance of joint homeostasis	Inhibit chronic inflammation and prevent exacerbation of arthritis [55,57,58]	-
	MerTK^+^ Macrophages	Express LYVE1, TREM2; potent phagocytic and pro-resolving functions	Maintain synovial homeostasis and promote sustained remission after drug tapering [6,65]	Proportion > 47.5% is significantly associated with sustained remission after dose reduction [65]
	PD-L1^+^ Macrophages	Express MerTK, secrete IL-10, inhibit inflammation via efferocytosis	Exert a protective role in the joint during arthritis, dampening immune responses [5]	Their immunoregulatory function is suppressed by IFN-γ in the RA joint [6]
	M2-like Macrophages (CD206^+^, CD163^+^)	Secrete IL-10, Arg-1; promote tissue repair and induce Treg cells	Exert anti-inflammatory, reparative, and immunoregulatory functions; their impairment contributes to disease progression [5,22,24,30,31]	Reduced proportions correlate with higher disease activity and radiographic joint destruction [24]
Pathogenic Macrophages	M1-like Macrophages (HIF-1α^+^)	Secrete TNF-α, IL-1β, IL-6, MMPs; promote angiogenesis	Drive synovitis, bone erosion, and cartilage degradation; positively correlated with disease activity [22,25]	Infiltration density positively correlates with DAS28 and radiographic erosion scores [22,25]
	Myeloid-derived Macrophages (e.g., CD40^+^CD206^+^CD163^+^)	Aberrantly infiltrate and differentiate into pro-inflammatory phenotypes, releasing destructive cytokines	Exacerbate arthritis; associated with disease activity and treatment response [32,49]	This dominant subset is associated with disease activity and therapeutic outcomes [60]
	SPP1^+^ Macrophages	Express osteopontin; exhibit pro-inflammatory and pro-fibrotic properties	Activate synovial fibroblasts, promoting invasiveness and collagen deposition [6,67]	Increased SPP1/SCARA5 ratio indicates treatment resistance and suggests poorer prognosis [67]
	STAT1^+^CXCL10^+^ Macrophages	Interferon-responsive; express CXCL10	Mediate Th1 cell chemotaxis and sustain chronic inflammation [66]	Contribute to Th1-driven chronic synovitis [66]
	IL1B^+^FCN1^+^HBEGF^+^ Macrophages	Express IL-1B, FCN1, HBEGF	Closely associated with early invasive lesions and bone destruction [66]	Enriched in early erosive lesions and linked to bone damage [66]
Functional Conflict and Microenvironment Regulation	M1/M2 Polarization Imbalance	M1 dominance coupled with M2 functional impairment and altered ratio	Leads to collapse of immune homeostasis and perpetuation of inflammation [20,32,58]	Imbalance correlates with persistent disease activity [58]
	Metabolic Reprogramming Dysregulation	M1 relies on glycolysis; M2 on oxidative phosphorylation; HIF-1α inhibits CPT1A	Promotes M1 polarization and suppresses M2 function [30,31]	-
	Extracellular Vesicle-mediated Communication	Macrophage-derived sEVs deliver miR-100-5p, activating the mTOR pathway	Promotes synovial fibroblast proliferation and inflammatory factor release [52,53,54]	-
	Hypoxic Microenvironment	HIF-1α upregulation impairs antigen presentation and stabilizes the M1 phenotype	Enhances the pro-inflammatory state and promotes joint destruction [21,26,27,35,36]	HIF-1α-driven metabolic shift promotes M1 polarisation and correlates with severity [21,26]
Therapeutic and Prognostic Relevance	Proportion of MerTK^+^ Macrophages	>47.5% associated with sustained remission	Can serve as a tissue-resident biomarker for predicting persistence of remission [65]	As above (MerTK^+^ row)
	Proportion of SPP1^+^ macrophage/SCARA5^+^ macrophage	Increased ratio correlates with treatment resistance	Indicates activation of fibrotic pathways and suggests poorer prognosis [67]	As above (SPP1^+^ row)

## Data Availability

The data that support the findings of this study are available from the corresponding author upon reasonable request.

## Abbreviations

RA	rheumatoid arthritis
ACPA	anti-cyclic citrullinated peptide antibody
Arg-1	arginase 1
CPT1A	carnitine palmitoyl transferase 1A
FLS	fibroblast-like synoviocytes
HIF-1α	hypoxia-inducible factor-1α
HMGB1	high mobility group box 1 protein
LXR	liver X receptor
MMPs	matrix metalloproteinases
NAMPT	nicotinamide phosphoribosyltransferase
RF	rheumatoid factor
sEVs	small extracellular vesicles
SSc	systemic sclerosis
